# Cofopose: Conditional 2D Pose Estimation with Transformers

**DOI:** 10.3390/s22186821

**Published:** 2022-09-09

**Authors:** Evans Aidoo, Xun Wang, Zhenguang Liu, Edwin Kwadwo Tenagyei, Kwabena Owusu-Agyemang, Seth Larweh Kodjiku, Victor Nonso Ejianya, Esther Stacy E. B. Aggrey

**Affiliations:** 1 School of Computer & Information Engineering, Zhejiang Gongshang University, Hangzhou 310018, China; 2 School of Information & Software Engineering, University of Electronic Science & Technology of China, Chengdu 611731, China; 3 Department of Computer Science, Kwame Nkrumah University of Science and Technology (KNUST), Kumasi 03220, Ghana

**Keywords:** DETR, human pose estimation, conditional DETR, convolutional neural network (CNN), detection

## Abstract

Human pose estimation has long been a fundamental problem in computer vision and artificial intelligence. Prominent among the 2D human pose estimation (HPE) methods are the regression-based approaches, which have been proven to achieve excellent results. However, the ground-truth labels are usually inherently ambiguous in challenging cases such as motion blur, occlusions, and truncation, leading to poor performance measurement and lower levels of accuracy. In this paper, we propose Cofopose, which is a two-stage approach consisting of a person and keypoint detection transformers for 2D human pose estimation. Cofopose is composed of conditional cross-attention, a conditional DEtection TRansformer (conditional DETR), and an encoder-decoder in the transformer framework; this allows it to achieve person and keypoint detection. In a significant departure from other approaches, we use conditional cross-attention and fine-tune conditional DETR for our person detection, and encoder-decoders in the transformers for our keypoint detection. Cofopose was extensively evaluated using two benchmark datasets, MS COCO and MPII, achieving an improved performance with significant margins over the existing state-of-the-art frameworks.

## 1. Introduction

Human pose estimation has long been a compelling yet challenging problem. Fundamentally, human pose estimation [1,2] concerns discovering the configuration of the body parts of a human from either consecutive images or single images. In computer vision, human pose estimation has necessitated a paradigm shift due to its practical importance for behavioral analysis, video surveillance [3], autonomous driving, human– computer interactions [4], healthcare, computer animation, skeleton action recognition [5], and video retrieval [6]. Recently, deep convolutional neural networks (DCNNs) have proven their capacity for visual understanding [7,8] and recognition [9,10] in terms of capacity and efficiency in human pose estimation. However, pose estimation is inherently challenging due to the ways that body shape changes in motion, inter- or intra-person occlusion, and background clutter. Recent pose estimation approaches can be roughly categorized into two groups: heatmap-based and regression-based approaches. Heatmap-based approaches adopt handcrafted features and heuristic pre/post-processing to encode an image to heatmaps, and then decode these heatmaps to predict keypoints. However, this approach faces challenges such as difficulties in updating and adaption. Regression-based approaches instead perform regression for the keypoints directly, entailing fewer intermediate stages and specifications. Although their performance is not on par with the heatmap-based approaches, they can be made end-to-end and readily integrated with other downstream tasks. In addition, regression-based approaches also aim to remove unnecessary designs by making the training objective and target output direct and transparent. Inspired by the recent regression-based method for pose estimation [11], we present a two-stage transformer approach for 2D pose estimation. Specifically, we use cascading transformers, consisting of a person detection transformer and a keypoint detection transformer. Cofopose differs from existing approaches insofar as it consists of conditional cross-attention, conditional DETR, and the use of encoder-decoders in the transformer architecture to achieve person and keypoint detection. That is, we leverage conditional cross attention and conditional DETR for person detection, and use encoder-decoders in the transformer to detect the keypoints. Cofopose shows competitive results in pose recognition compared with the existing regression-based and heatmap-based methods. In brief, the contributions of our work can be summarized as follows:

- We propose Cofopose, a two-stage approach consisting of person- and keypoint-detection transformers for 2D human pose estimation.
- Cofopose comprises conditional cross-attention, conditional DETR, and encoder-decoders in the transformer framework to achieve person and keypoint detection. Specifically, we utilize conditional cross-attention and fine-tuned conditional DETR for our person detection, and encoder-decoders in the transformers for our keypoint detection.
- Cofopose achieves state-of-the-art accuracy on both the MPII and MS-COCO benchmark datasets. Furthermore, the contributions of the hypothesized architecture have been confirmed using ablation investigations.

## 2. Related Work

### 2.1. Transformers

Machine translation has greatly benefited from the introduction of attention [12], specifically transformer models, which have significantly improved the performance of deep learning architectures when performing language-based tasks [13]. The popularity of transformers has recently increased because of vision-related issues [14]. These methods of attention gather data from a long input sequence and divide it up into their constituent parts. Recently, their application has been expanded to include speech recognition [15,16] and generative language modeling [17], among numerous other tasks [14,18]. In recent years, various architectures have been proposed to solve computer vision issues such as object detection [14,19], pose estimation [11,20], low-level image processing and classification [21,22], and transformer transducer [23] tasks via features extracted from a CNN’s backbone. By learning the weight of each node, researchers propose a graph attention network (GAT) [24] that combines neighbor information using self-attention. Akbari et al. [25] introduced the model Video-Audio-Text Transformer (VATT) for generating multi-modal representations from unprocessed text, video, and audio. VATT further investigates the relationship between the frame sequences, audio, and sentences. Similarly, Huang et al. [26] argue that, through the power of self-attention, the transformer-encoder block can be used to upscale the component of sequences of keypoints. For some time now, video–text representations have been learned by using global action and local regional objects [27] as inputs; to improve communications between various sources. Detection with Transformers (DETR) [28] formulates the object detection architecture to predict a box set in order for the detection model to perform end-to-end training. Both 2D pose estimation and 3D human pose estimation [27] applications can benefit from transformers.

### 2.2. Human Pose Estimation

Human pose estimation can be either image based or video based. With image-based learning, Kortylewaki et al. [29] proposed an architecture that uses the VGG-16 base model to learn the correlations between human body parts. Furthermore, probability heatmaps [30] are deployed to identify the locations of joints. Zhang et al. [31] made significant progress using multi-scaled feature pyramids for human pose estimation. In a practical sense, all of these achievements were accomplished by deploying either a bottom-up or a top-down strategy. As might be expected, the bottom-up approach [32,33] extracts each human body part and then integrates them to form a full person. In contrast, the top-down approach executes person detection at the initial stage and then accomplishes single-person human pose estimation for each body part. To accomplish high-resolution feature maps, Sun et al. [8] suggested a Deep High-Resolution Network (HRNet) for multi-scale fusion. This improvement seeks to address the problem encountered in keypoint heatmaps regarding spatial precision. On the other hand, video-based pose estimation can be boosted by capturing temporal information together with appearance information across frames. Many prior models [34,35] address video-based pose detection tasks as a two-sided problem, comprising (1) identifying the keypoints that exist in individual frames, and then (2) using temporal smoothing techniques. In addition, the tracking of human poses [36] has been widely adapted to simultaneously refine human pose estimation. Tao et al. [37] suggested convolutional LSTMs for capturing temporal and spatial context information, while others utilize Recurrent Neural Networks (RNN) [38,39]. Raaj et al. [40] proposed an algorithm for pose detection using a warping mechanism on the PoseTrack datasets. Liu et al. [41] proposed a DC-Pose that uses HRNet [8] as the backbone for pose detection, while tracking through the novel Pose Temporal Merger (PTM) and Pose Residual Fusion (PRF), as well as Pose Correction Network (PCN), on Pose-Track datasets, to achieve results that were significantly better than the existing state-of-the-art. Zhang et al. [42] also provided a distribution-aware coordinate representation to cope with the quantization error of down-sampling heatmaps. In addition to being tolerant of jitter errors, this type of learning schema is also tolerant of spatial ambiguity in its implementation. Whenever coordinate migration refinement is used as a post-processing step, the likelihood of false positives is reduced. Luvizon et al. [43] suggested an end-to-end model architecture based on regression for human pose estimation, using the soft-argmax function to extract feature maps into keypoint coordinates of a complete differentiable model. A summary of some related works is also presented in Table 1.

## 3. Model

### 3.1. Revisiting Conditional DETR

First, let us revisit conditional DETR architectures, as this will be beneficial for us when establishing effective architectures for pose estimation. A typical conditional DETR comprises of a number of encoder and decoder blocks, a CNN backbone, and object class predictors as well as box position predictors. Self-attention and feed forward are the two major components of the encoder, which is designed specifically to enhance the quality of the content embeddings that are produced by the CNN backbone. The decoder layer comprises three parts: (i) a self-attention layer for eliminating duplication prediction, which interacts with the embeddings output from the last decoder layer, and is then used for class and box prediction; (ii) to enhance class and box prediction, cross-attention layers are used to aggregate the embedding output from the encoders and fine-tune the embedding output from the decoders; and (iii) feed-forward layers.

Furthermore, a multi-head attention technique [12] has been proposed to describe the difficult relationships between token entities from many perspectives. It uses multiple heads in order to model attention from distinct representation sub-spaces and positions simultaneously. Technically, a standard cross-attention has *q*: queries, *k*: keys, and *v*: values, by notation:

(1) $$MHA_{q,k,v}=Concat\left(H_{i}\ldots ,\;H_{m}\right)W^{O}$$

where *MHA* represents the multi-headed attention.

(2) $$H_{i}=Soft\left(\frac{qW_{i}^{q}{\left(kW_{i}^{k}\right)}^{T}}{\sqrt{d_{k}}}\;vW_{i}^{v}\right)$$

where $d_{k}$: dimension of the key, m: number of heads, ($W_{i}^{q}$, $W_{i}^{k}$, $W_{i}^{v}$): linear projections, and $W^{O}$: projection matrix for combining the various features heads. Each key is constructed by joining a content key $C_{k}\;$(the output encoder content embedding) with a spatial key $P_{k}$. (the positional embedding of the matching 2D coordinate). The said value is generated from the content embedding, the same as the content key and the encoder output. In the original DETR, a query is generated by aggregating a content query $C_{q}\;$(for embedding the outcome produced by the decoder’s self-attention) together with a spatial query $P_{q}$ for object query $O_{q}$. Here, *N* consists of 100 object queries, with *N* queries: each query produces a candidate detected output in a single decoder layer. In contrast to existing DETR cross-attention mechanisms, the conditional cross-attention technique partitions the roles of content as well as spatial queries, such that spatial queries concentrate on the spatial while content queries focus on content attention weights. Additionally, the computation of the spatial query $P_{q}$ from the embedding of the previous decoder layer is another important task.

### 3.2. Cofopose Architecture

**Figure 1:**
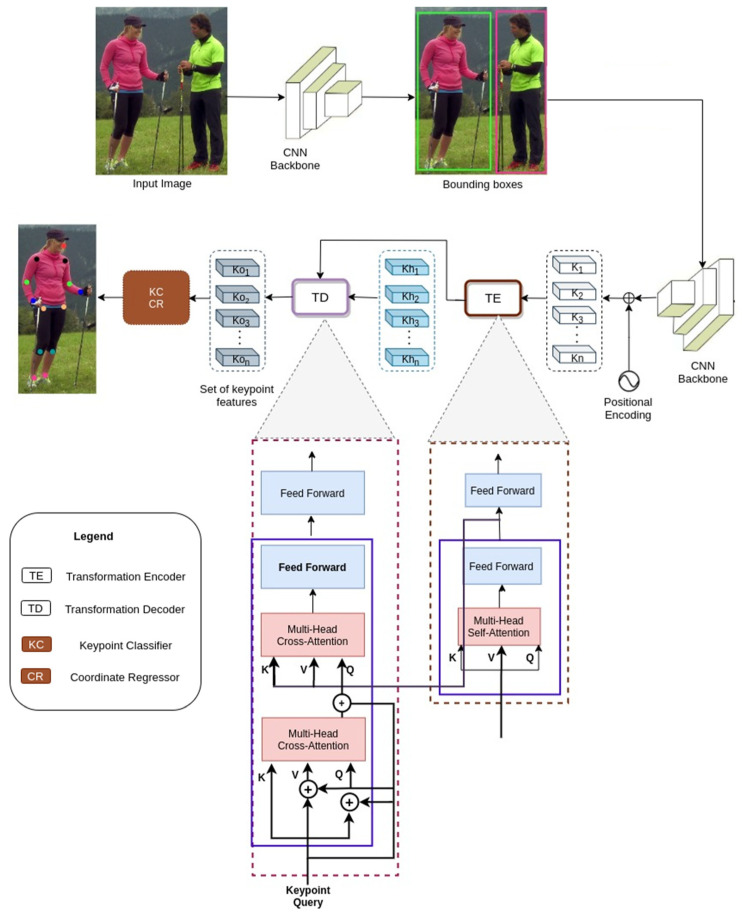
Detailed architectural pipeline of the proposed Cofopose. It starts by extracting and detecting the keypoint positions using the backbone net. Afterwards, the feature map is fed forward. Finally, the feature map is concatenated together with the keypoints so that the encoder can encode their appearances with the 2D locations, and give them as inputs to the transformer decoder in order to predict the human poses.

Drawing inspiration from [11] and tapping into the power of multi-cross attention [12], we propose Cofopose, a two-stage architecture for human pose estimation. In the initial stage, the end-to-end detector predicts all the input images for the person detector; then, the output of the person detection transformer is leveraged as an input for the keypoint detection transformer. Intuitively, the model comprises a backbone, a transformer encoder and decoder, a classifier, and a regressor for boxes and coordinates, as shown in Figure 1. The regression transformer’s task is to perform direct learning and inference, in this case by obtaining complex keypoint correlations between input and output images, estimating queries via conditional cross-attention, and modeling the conditional probability via self-attention.

#### 3.2.1. Transformer Encoder

The transformer encoder tries to enhance the output of the CNN backbone’s information embeddings. It comprises a stack of numerous encoder layers, each of which consists primarily of a self-attention layer and a feed-forward layer. The encoder phase flattens and feeds the image features constructed by the network (CNN) into a transformer encoder, to provide context-specific image features.

#### 3.2.2. Transformer Decoder

The transformer decoder comprises a series of decoder layers stacked on top of one another. It comprises: a self-attention layer for deleting duplication prediction, which interacts with the embeddings produced by the former decoder layer and is utilized for class regression as well as box regression; a cross-attention layer that aggregates the encoder’s embeddings in order to further enhance the decoder’s embeddings for improved classification; and box and keypoint regression and multi-layer perceptrons (the feed-forward layer) which transform the output of one attention layer in order to make it fit better with the input of the subsequent attention layer as the main components. When given a fixed set of learned query embeddings as input, the transformer decoder determines the differences between objects using image features as context, and outputs all object queries concurrently. For the box regression, similar to DETR [28], the decoder layer embedding estimates the candidate bounding box ($C_{b}$), formulated as:

(3) $$C_{b}=\sigma \left(\left(D_{e}\right)+{\left[u^{T}\;\;0\;0\right]}^{T}\;\right)$$

where $C_{b}$: four-dimensional vector for candidate bounding box ${\left[b_{cy}\;b_{w}\;b_{h}\right]}^{T}$, *σ*: sigmoid function for predicting $C_{b}$ within the range of [0, 1], ∮ (): feed-forward network for estimating the unnormalized box, $D_{e}$: decoder embedding, $u^{T}$: 2D unnormalized coordinate to be used as a reference point in contrast to DETR, which is (0, 0). Two options are key here: either to learn the reference point u as a parameter for estimating individual candidate bounding boxes, or by corresponding query generation.

#### 3.2.3. Conditional Cross-Attention

The suggested conditional cross-attention method constructs the query by joining the content query $C_{q}$, which is output from the decoder’s self-attention, with the spatial query $P_{q}$. Thus, the key is generated by concatenating the content key $C_{k}$ with itscorresponding spatial key $C_{k}$. In this way, cross-attention weights can be simulated as content attention and spatial attention weights. The weights are calculated by taking the dot products of content and spatial information, which are denoted as:

(4) $$C_{q}^{T}C_{k}+P_{q}^{T}P_{k}$$

In contrast to the traditional DETR cross-attention procedure, the system now segregates the responsibilities of spatial and content queries, with spatial queries focusing on the spatial as well as content attention weights, accordingly. Additionally, the spatial query $P_{q}$ is computed from the prior decoder embedding layer $D_{e}$, thus establishing that the spatial information of separate regions is influenced by a combination of both decoder embedding ($(D_{e}$) and a reference point (u). Finally, they are mapped to their corresponding embedding space by constructing the query $P_{q}$, so that the spatial query is located in the same space as the keys’ 2D coordinates. The conditional spatial query prediction tries to estimate the resultant conditional spatial query produced by the decoder embedding ($(D_{e}$) and the reference point u:

(5) $$(D_{e},\;u)\Rightarrow P_{q}$$

It does so by mapping the positional space to which the keys’ normalized 2D coordinates are matched. This adjusts the reference point u before matching it to a 256-dimensional sinusoidal positional (space) embedding, in the same manner as the keys represented:

(6) $$P_{u}=\psi \left(\sigma \left(D_{e}\right)\right)$$

ψ represents sinusoidal positional (space) embedding. Here, the displaced data embedded in the decoder embedding $D_{e}$ is mapped to a linear representation in the same positional space via an FFN (∮), comprising learnable linear projection, ReLU, and learnable linear projection concatenated together: T = $\oint^{\;}\left(u\right)$.

#### 3.2.4. Keypoint Detection

An image *I* with a four-dimensional vector for candidate bounding box ${\left[b_{bx\;}\;b_{cy}\;b_{w}\;b_{h}\right]}^{T}$ output from the person-detection transformer is cropped and fed into the backbone convolutional neural network, $I\in R^{B\;*\;3\;*\;H\;*\;W}$, where B: input batch size, 3: color channels, and H, W: height and width of the image, as shown in Figure 1. In the initial stage, the keypoints that are most likely to correlate to body joint locations are first identified and extracted. A series of computation and downsampling steps are performed by the backbone net to obtain lower-resolution feature maps, which are denoted by the letters F∈ R^B X CXH/SXW/S^, with S representing stride. Due to the fact that mapped queries are necessary for computing loss for transformer keypoint detection, the mismatched tokens are eliminated. It is valuable to know that, in this phase, the earlier encoder-decoder transformer predicts in a parallel manner with the former. In the final stages, a classifier tries to predict among *N* categories of joints (*N* = 17, in MSCOCO) with a background and a two-channel regression head to yield the coordinates of each individual joint. The proposed architecture produces a predetermined number of more predictions than the ground truth *N*. It is necessary to find a mapping between them so as to compute the loss. The training target and loss function were established as a bipartite matching problem, a similar approach to that seen in DETR [28]. We use the Hungarian algorithm to discover the best bipartite matching between both the predicted and ground-truth entities and, as a result, defined the cost for computing and back-propagating the gradients. We attempted to deduce a matching cost (*L*) with the optimized loss as denoted below:

(7) $$L_{\varepsilon }=\underset{\sigma }{\mathrm{argmin}}=\Sigma_{i}^{N}\;L_{\varepsilon }\left(y_{i},\;{\hat{y}}_{\sigma \left(i\right)}\;\right)$$

*σ*(*i*) denotes the regression to be mapped with the number of joints. Queries are correlated by adopting a mixture of conditional probability classifications, as well as the joint deviation. The joint loss function with its corresponding query *σ(i)* is formulated as:

(8) $$L_{\varepsilon i}=-{\hat{P}}_{\sigma \left(i\right)}\;\left(L_{\varepsilon i}\right)+\mathrm{II}\;b_{1}-{\hat{b}}_{\sigma \left(i\right)}\;\mathrm{II}$$

*σ*(*i*) shows the probability class of the corresponding query and $L_{\varepsilon i}$ represents the label class for *i*-th joints or keypoints; as such, the final architectural loss function can be estimated by changing the probability $-{\hat{P}}_{\sigma \left(i\right)}\;\left(L_{\varepsilon i}\right)$ to the negative log-likelihood $-log{\hat{P}}_{\sigma \left(i\right)}\;\left(L_{\varepsilon i}\right)$ for the mapped queries. When a mismatch is detected, queries are backpropagated for classification loss. This disparity with the class can be corrected by assigning weight 0.1 to the log-probability. At this stage, the conditional spatial query is calculated by converting the embedded space reference point: $P_{q}=T_{pu}$. An easy approach with lower computational costs is adopted for better accuracy, with φq representing a 256-diagonal elements vector. The conditional spatial query $\left(P_{q}\right)$ is formulated by performing element-wise multiplication of both sides, as denoted below:

(9) $$P_{q}=T_{pu}=\;\phi_{q}\;⊗\;\;P_{u}$$

## 4. Experiments

### 4.1. Setup

**Dataset**: We analyze our proposed model with reference to the difficult MS COCO object-detection benchmarks [54], utilizing the regular practice settings. The dataset contains over 160 K photos that have been culled from the web and organized into 80 main categories. In addition, the dataset is divided into three subgroups: train2017, val2017, and test2017, which comprise 118 K images, 5 K images, and 41 K images, respectively. For pose estimation, the COCO dataset has about 200,000 photos of over 150,000 people labeled with up to 17 keypoints of annotation. The dataset is divided into three sets: the train set, the validation set, and the test-dev set, which contain 57 k, 5 k, and 20 k images, respectively. For easy comparison with the state-of-the-art designs, we conducted training using the training images (including humans) and reported the findings for the validation set and also on the test set. The conventional mean average precision (mAP) was adopted to report the accuracy of the Cofopose. Additionally, we deployed the COCO standardized Object Keypoint Similarity (OKS), defined as:

(10) $$OKS=\underset{i}{\sum }\frac{exp\left(-\frac{d_{i}^{2}}{2s^{2}k_{i}^{2}}\right)\delta \left(v_{i}>0\right)}{\delta \left(v_{i}>0\right)}\;$$

Thus, given the 17 annotated keypoints *i* ∈ {1, 2, 3, 4, …, 17}, the Euclidean distance between the predicted keypoint and its related ground truth is denoted as *d_i_*, *v_i_*: visibility of the ground truth, *s*: the object scale, *k_i_*: the COCO constant, and α is 1 when the visibility is positive and zero for negative visibility. Additionally, following the normal metrics for the COCO dataset, we computed the mean average precision and recall. Average precision: AP50, AP75, APS (Small), APM (Medium), and APL (Large). The recall score was performed at AR50, AR75, ARS (Small), ARM (Medium), and ARL (Large). For the purposes of comparison with other approaches, we primarily used the average precision (AP) metric, which is the key challenge metric in COCO, as well as FLOPs, and evaluates the computing overhead. Moreover, we also conducted an extensive experiment on MPII [55]. There are roughly 25 k photos and about 40 k people with 16 joint labels represented in the MPII dataset. All input photos are cropped in accordance with conventional training settings [8,51] in order to provide fair comparisons. For training, we randomly divided the data into two portions for the backbone architecture search: 80% for operating weight training and 20% for updating the model architectural parameters.

### 4.2. Model Settings

We utilized the AdamW optimizer [56] during model training. The baseline learning rate for the ResNet backbone was set to 1 × 10^−5^ whereas both the remaining part and the weight decay were set to 1 × 10^−4^. The dimensions of the input image snippet setting were 384 × 288 or 512 × 384 for COCO, and 384 × 384 or 512 × 512 for MPII. We used the default parameters for HRNet [8] and Simple Baseline [51]. For the person detection transformer, we began by adapting the method [11] for tuning a person detector using weights developed by DETR [28].

### 4.3. Implementation Details

We employed the top-down human pose estimation (HPE) methodology outlined in [7,8,10] and a hybrid approach comparable to Li et al. [11]. Here, a person detector initially detects the instance of a single person, and thereafter keypoints are estimated. γ1 and γ2 are set to 0.9 and 0, respectively. The following techniques are used: data augmentation, random rotation ([−40, 40]), random scaling ([0.7, 1.3]), and flipping, as well as half-body data augmentation. The setting for the transformer section is as follows: the number of encoder layers—6, the number of decoder layers—6, keypoint queries—100. Transformers have the dropout rate reported by Li et al. [11]. Similarly to Smith et al. [21], after 40 epochs for 50 training epochs, the learning rate is reduced by a factor of 10. The training procedure stops after 50 epochs for both the COCO and MPII datasets. The primary architectural novelty in this research is that we use conditional spatial embeddings, like those of spatial queries, for conditional multi-head cross-attention; moreover, we integrate the spatial and content queries (keys) via concatenation rather than addition. Since there are no decoder content embeddings in the first cross-attention layer, we use the DETR-version [28] modification: we merge the positional embedding estimated from the object query into the original query (key).

### 4.4. Comparism with Existing State-of-the-Art Archectures

In this section, we evaluate Cofopose against existing state-of-the-art 2D pose estimation algorithms using MPII, COCO validation split, and COCO test-dev split. We compare these methods based on accuracy, convergence, and computational cost.

**Results from MPII**: In Table 2, we show the performance comparisons of Cofopose with state-of-the-art (SOTA) models, as well as the performance gain. We show the outcome from simple baseline (SBL) [51], CPM [46], PETR [11], and our proposed architecture using the MPII dataset. With 50 training epochs, PETR achieves low accuracy in comparison with when the training setting is set to 200 epochs. With the Cofopose architecture, just 50 epochs of training using ResNet-101 and ResNet-152 as the model backbone achieves slightly better or equivalent results compared with SBL, CPM, and PETR with 200 epochs of training. In addition, we trained PRTR [11] and Cofopose for 50 epochs with the same settings for easy comparison. The results that are in bold show our best model, and the one that is underlined is the best SOTA model.

**Results for COCO:** In Table 3, we compare our proposed Cofopose with other pose estimation architectures using the COCO 2017 validation set. In Table 4, we look at how our proposed Cofopose compares to other state-of-the-art pose estimation networks on the COCOtest-dev set. H-B*** and R-B*** denote heatmap-based keypoint heatmap prediction and post-processing to decode coordinates, and regression-based direct keypoint coordinate prediction, respectively. #Params and FLOPs are computed for the pose estimation model, but not for detection and tracking or grouping of keypoints. The results that are in bold show our best model and the ones that are italicized and underlined represent the best SOTA model for both heatmap-based and regression-based approaches.

***On validation set***, Cofopose outperforms various heatmap-based approaches with the same backbone (Res-50, CPN [50]) (71.9 AP as compared to 68.6 AP). Cofopose with the Res-101 backbone is analogous to PointSetNet [58] for the validation set, which has a more sophisticated backbone (HRNet-W48). Even Cofopose with Res-101 shows an improvement of 7% as compared to the heatmap-based Hourglass-8 stacked approach [47], and a largely similar result with SB [51] using the same Res-101 as the backbone. Furthermore, Cofopose outperforms a number of regression-based approaches, such as PointSetNet [58] (74.2 AP as compared to 65.7 AP) and PRTR [11] (74.2 AP as compared to 73.3 AP). It is noteworthy that, compared to CPN [50], Cofopose with a Res-50 backbone achieves low computational costs (10.2 GFLOPs) relative to its heatmap-based counterparts on the validation set. Our performance gain, as compared to the best regression-based and heatmap-based SOTA, is +0.9 and +0.6 AP, respectively, which is significant.

***On test-dev***, as demonstrated in Table 4, Cofopose achieves the greatest outcome among the heatmap-based approaches. Cofopose using six encoder layers with Res-101 produces 71.5 AP, which is superior to its heatmap-based counterparts PifPaf [49] (65.5) and PersonLab [59] (65.5 AP), and its regression-based counterparts DirectPose [60] (63.3 AP) and Integral [11,57] (67.8) with the same backbone. Cofopose achieves the best minimal computational costs (18.3 GFLOPs) as compared to the best heatmap-based models (32.9 GFLOPs). Our best performance with the HRNet-32 backbone on dev-test is comparable with both the best heatmap-based and regression-based methods. It is noteworthy that there is a slight increase in AP over PRTR [11], demonstrating that Cofopose can identify more precise keypoint coordinates. In particular, Cofopose’s findings with 50 epochs are comparable to the best-published pose estimation results, such as PRTR [11], SB [51], HigherHRNet [51], Dark [42], and SPM [61] with 200 epochs. Our performance gain as compared to the best regression-based SOTA is +2.0 AP, which is a significant improvement and demonstrates that our network is comparable to its heatmap-based counterparts.

### 4.5. Ablation Study

**Figure 2:**
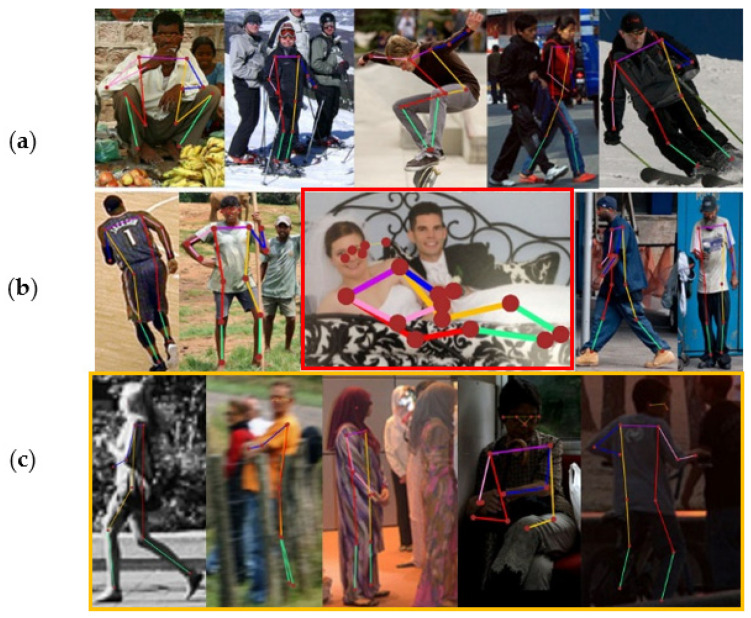
Visualization of the pose estimation results of Cofopose using some image samples from the MS-COCO dataset. Rows (**a**,**b**) are results estimated from images with good illumination, with the exception of the picture with the red border from row (**b**), which is occluded by clothes. Even though the image with the red border looks more occluded, Cofopose was able to estimate the pose accurately; (**c**) shows Cofopose results on blurred and low-illumination images.

**Figure 3:**
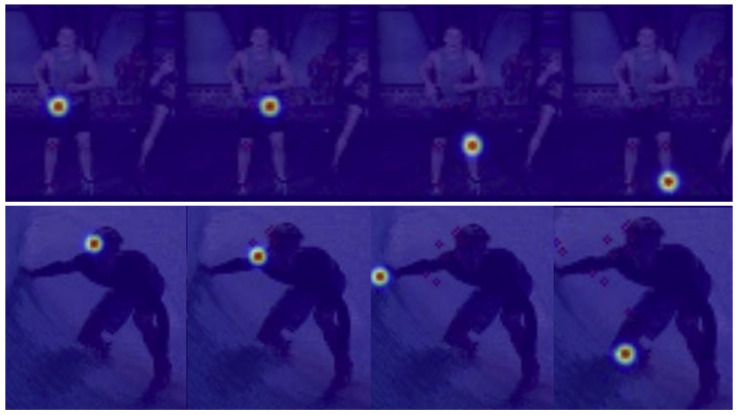
Visualization of Cofopose’s process of decoding for the keypoint detection transformer on MPII and COCO is denoted by the first and second rows, respectively. For MPII, the first, second, third, and fourth columns represent the right hip, left hip, left knee, and left ankle, respectively. For COCO, the first, second, third, and fourth columns represent right eye, right shoulder, right wrist, and right knee, respectively.

**Figure 4:**
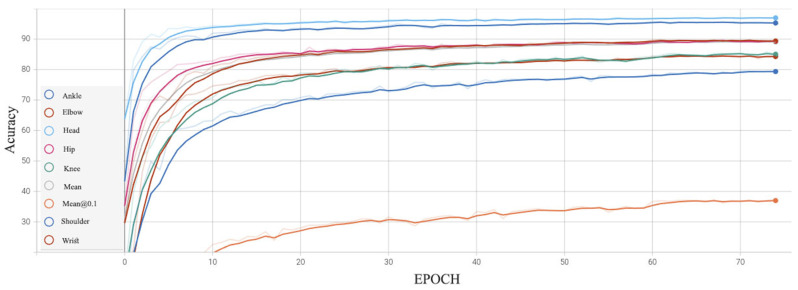
Cofopose trade-off accuracy speed for keypoint detection with ResNet-101 as a backbone prowith a higher resolution of 512 × 512 for 75 epochs, when used on the MPII dataset.

**Figure 5:**
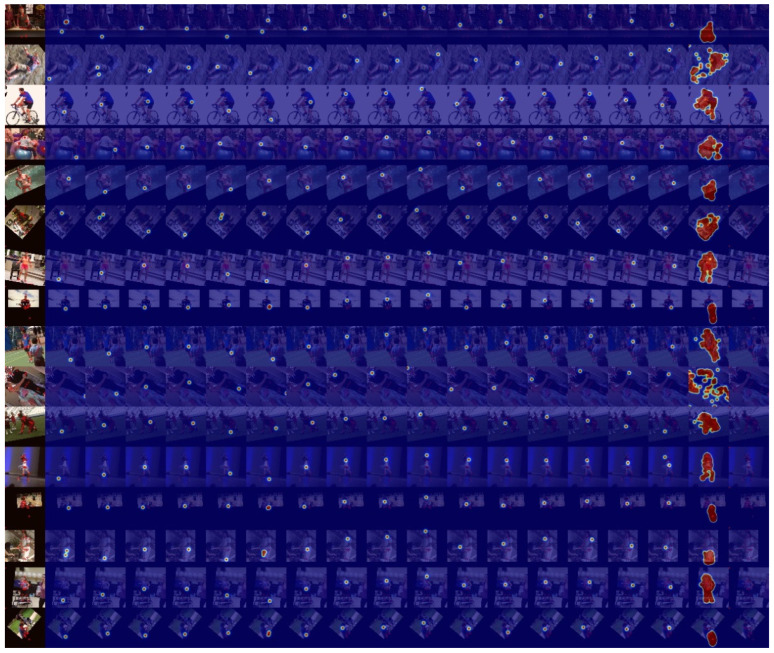
Visualization of Cofopose’s process of decoding for the keypoint detection transformer. Each row shows an overlay of heatmaps of 100 queries for all individual keypoints.

In our experimental settings, pose samples acquire prior knowledge and information by learning the statistical significance of keypoints from the dataset. To represent the embedded information, we compute and display the inner product matrix, as shown in Figure 2. It can be observed from rows (a) and (b) that Cofopose is robust in both low and high illumination. In addition, in Figure 2 row (b), in the picture with the red border, we illustrate how Cofopose performs on occlusion with an image occluded by clothes. The results show that Cofopose is also robust to occlusion. Row (c) shows Cofopose used on blurred and low-illumination images. The first three images are blurred images demonstrated on Cofopose and the last image shows Cofopose on low illumination images. In summary, Cofopose is able to overcome some existing challenges, such occlusion, low illumination, and blurry images, and achieves superior results. In Figure 3, we visualized Cofopose’s process of decoding for the keypoint detection Transformer on MPII and COCO. In the first row, the first column, the second column, the third column, and the fourth column represent the right hip, left hip, left knee, and left ankle, respectively, on the MPII-dataset. For COCO, the first column, second column, third column, and fourth column of the second row denote the left eye, right shoulder, right wrist, and right knee, respectively. In Figure 4, we present the graphical trade-off accuracy speed for each keypoint, with ResNet-101 as a backbone, on the MPII dataset, with the head and shoulder obtaining the highest accuracy. Figure 5 depicts Cofopose’s process of decoding for the keypoint detection Transformer. Each row shows an outline of heatmaps of 100 queries for all individual keypoints. In Table 5, we compare the efficiency of Cofopose to other state-of-the-art variants, where we established that Cofopose achieves a competitive speed/accuracy trade-off.

## 5. Conclusions

In this paper, we introduced a two-stage 2D human pose estimation method that uses person- and keypoint-detection transformers; it is named Cofopose. Cofopose consists of conditional cross-attention, conditional DETR, and encoder-decoders in the transformer architecture to achieve person and keypoint detection. Specifically, we use conditional cross-attention and conditional DETR for person detection, and encoder-decoder transformers for regressing their keypoints. Furthermore, we demonstrate the dissemination of keypoint queries in a variety of ways in order to reveal the transformer′s internal mechanism for gradual detection refinement. Ablation experiments also show the effectiveness of our proposed model during inference. Cofopose was extensively evaluated on two benchmark datasets, MS COCO and MPII; with less training, it achieved an improved performance (with significant margins of +2.0 for the COCO dev set, +0.9 for the val set, and +0.6 for MPII) over the top regression-based state-of-the-art methods.

We believe that our study will serve as a foundation for future research in this crucial area. Future work will focus on enhancing the human pose estimation architecture and developing a new architecture for multi-person pose estimation. The datasets utilized in this work emphasize frame-by-frame inference; thus, there is an opportunity to develop video estimate techniques that give more temporally consistent [41,65] results.

## Figures and Tables

**Table 1:** Summary of some related works, and their contributions and limitations.

Models	Remarks	Limitations
DeepPose [44]	A model was created to study the results of jointly training a multi-staged framework with repeated intermediate inspection.	Regressing to a location is extremely difficult, increasing the complexity of the learning and reducing generalization.
ConvNet Pose [45]	Proposed an architecture to generate discrete heatmaps instead of continuous ones	The architecture lacks structural modeling.
CPM [46]	Integration of the convolutional network into pose machines, allowing them to learn image features and image-dependent spatial models to estimate human poses.	Vulnerable when multiple individuals are nearby, computational cost, and, if the detection of individuals fails, there is no possibility of recovering.
Stacked-Hglass [47]	Utilized repeated bottom-up, top-down, and intermediate supervision to improve the network’s performance.	Hundreds of parameters, and loss functions become incredibly complex
DeeperCut [48]	Introduced strong body part detectors to produce effective bottom-up proposals for body joints, and utilized the deep ResNet for human pose estimation.	The pairwise representations are very hard to regress.
PAF [49]	Proposed a model to connect human body parts via Part Affinity Fields (PAF), a non-parametric method, to achieve bottom-up pose estimation.	Grouping body parts is very challenging when there is a large overlap between people.
CPN [50]	Proposed a CPN structure composed of GlobalNet and RefineNet. Easy keypoints are estimated by the GlobalNet, while the estimation of hard keypoints is performed by RefineNet.	High computational costs, and vulnerable when multiple individuals are nearby.
SB [51]	Introduced an intuitive and simplified architecture that is made up of few deconvolutional layers at the end of ResNet to estimate the keypoint heatmap.	High computational cost, and vulnerable when multiple individuals are nearby.
HRNet [8]	Proposed an innovative and intuitive method to keep a high-resolution representation throughout the process.	Fails to capture long-range interactions between joints, and has high computational complexity.
CFA [52]	Provides a cascaded multiple hourglass, as well as aggregating high, medium, and low-level features to better capture global semantic and local detailed information.	If the detection of individuals fails, there is no possibility of recovering, and it has a high computational cost.
occNet [53]	Revealed tow occlusion detection networks, namely Occlusion Net (OccNet) and Occlusion Net Cross Branch (OccNetCB), to perform pose estimation of all the detected persons.	Suffers from early commitment, hence, if the detection of an individual person fails, recovery becomes very difficult.
Dark [42]	The researchers identified the design limitations on the existing standard coordinate-decoding model, and introduced a principled distribution-aware decoding model.	Encounters the problem of sub-pixel localization.

**Table 2:** Comparisons on the MPII validation set (PCKh @0.5). Results for lower and higher resolutions with different backbones. We use * to denote Cofopose with lower resolution and ** for higher resolution, with 256 × 256 and 512 × 512, respectively.

Method	Backbone	Epoc	Head	Shou	Elbow	Wrist	Hip	Knee	Ankle	Mean
CPM [46]	CPM	200	96.2	95.0	87.2	82.2	87.6	82.7	78.4	87.7
SBL [51]	Res-152	200	97.0	95.9	90.3	*85.0*	89.2	85.3	*81.3*	89.6
Integral [57]	Res-101	200	-	-	-	-	-	-	-	87.3
PRTR [11]	HRNet-W32	200	*97.3*	*96.0*	*90.6*	84.5	*89.7*	*85.5*	79.0	89.5
	HRNet-W32	50	93.3	91.4	73.5	60.0	81.0	58.1	41.7	73.2
**Cofopose**	Res-101 *	50	96.0	94.2	84.3	75.8	86.9	78.0	71.1	84.6
	Res-101 **	50	97.6	95.8	90.5	84.9	89.8	85.1	79.1	89.6
	Res-101 **	75	**97.9**	**96.2**	90.3	**85.3**	**90.3**	**85.7**	**80.4**	**90.1**
	Res-152 *	50	96.8	94.5	85.2	77.3	88.8	78.8	73.4	85.6
	Res-152 **	50	97.1	95.5	88.6	82.3	88.6	82.5	75.5	87.9
	HRNet-W32 **	50	96.5	94.0	84.8	77.1	87.3	77.1	79.0	84.5
**Performance Gain**			**+0.6**	**+0.2**		**+0.3**	**+0.6**	**+0.2**		**+0.5**

**Table 3:** Comparisons for the COCO val set. H-B** and R-B** represent the heatmap-based approach and the regression-based approach, respectively.

Method	Backbone	Input	#Params	GFLOPs	AP	AP_50_	AP_75_	AP_M_	AP_L_	AR
H-B**										
8-stage Hglass [47]	Hglass-8 stacked	256 × 192	25.1 M	14.3	66.9	-	-	-	-	-
CPN [50]	Res-50	256 × 192	27.0 M	6.20	68.6	-	-	-	-	-
SB [51]	Res-50	384 × 288	34.0 M	18.6	72.2	*89.3*	78.9	68.1	79.7	77.6
SB [51]	Res-101	384 × 288	53.0 M	26.7	*73.6*	69.9	*80.3*	*79.1*	*81.1*	79.1
R-B**										
PointSetNet [58]	ResNeXt-101-DCN	-	-	-	65.7	85.4	71.8	-	-	-
	HRNet-W48	-	-	-	69.8	88.8	76.3	-	-	-
PRTR [11]	HRNet-W32	512 × 384	57.2 M	37.8	*73.3*	*89.2*	*79.9*	*69.0*	*80.9*	*80.2*
**Cofopose**	Res-50	384 × 288	39.2 M	**10.2**	69.3	89.4	76.3	64.0	77.1	76.9
	Res-50	512 × 384	40.4 M	17.7	71.9	90.4	79.1	67.3	79.9	79.1
	Res-101	512 × 3 84	59.3 M	32.3	73.1	**90.4**	80.3	68.4	80.8	80.1
	HRNet-W32	384 × 288	56.0 M	20.7	74.1	90.3	80.8	69.9	81.3	80.9
	HRNet-W32	512 × 384	56.0 M	36.9	**74.2**	90.2	**81.0**	**70.1**	**81.8**	**81.3**
Performance Gain(R-B**)					**+0.9**	**+1.2**	**+1.1**	**+1.1**	**+0.9**	**+1.1**
Performance Gain(H-B**)					**+0.6**	**+1.1**	**+0.7**		**+0.7**	**+2.2**

**Table 4:** Comparisons for the COCO test-dev set, with the exclusion of systems trained using external data. H-B*** and R-B*** represent the heatmap-based approach and the regression-based approach, respectively.

Method	Backbone	Input	#Params	GFLOPs	AP	AP_50_	AP_75_	AP_M_	AP_L_	AR
H-B***										
Mask-RCN [62]	Res-50	-	-	-	63.1	87.3	68.7	57.8	71.4	-
G-RMI [10]	Res-50	353 × 257	42.6 M	57.0	64.9	85.5	71.3	62.3	70.0	69.7
Assoc. Embe [63]	Hglass-4 stack	-	-	-	65.5	86.8	72.3	60.6	72.6	70.2
PifPaf [49]	Res-101	-	-	-	65.5	-	-	62.4	72.9	-
PersonLab [59]	Res-101	-	-	-	65.5	87.1	71.4	61.3	71.5	70.1
HigherHRNet [7]	HRNet-W48	-	-	-	70.5	89.3	77.2	66.6	75.8	74.9
CPN [50]	ResNet-Inception	384 × 288	-	-	72.1	91.4	80.0	68.7	77.2	78.5
SB [51]	Res-152	384 × 288	68.6 M	35.6	73.7	91.9	81.1	70.3	80.0	79.0
Dark [42]	HRNet-W48	384 × 288	63.6 M	32.9	76.2	92.5	83.6	72.5	82.4	81.1
R-B***										
CenterNet [64]	Hglass-2 stack	-	-	-	63.0	86.8	69.6	58.9	70.4	-
DirectPose [60]	Res-101	-	-	-	63.3	86.7	69.4	57.8	71.2	-
SPM [61]	Hglass-8 stack	384 × 384	-	-	66.9	88.5	72.9	62.6	73.1	-
Integral [11,57]	Res-101	256 ×256	45.0 M	11.0	67.8	88.2	74.8	63.9	74.0	-
PointSetNet [58]	HRNet-W48	-	-	-	68.7	89.9	76.3	64.8	75.3	-
PRTR [11]	HRNet-W32	512 × 384	57.2 M	37.8	*72.1*	*90.4*	*79.6*	*68.1*	*79.0*	*79.4*
**Cofopose**	Res-101	384 × 288	58.9 M	**18.3**	69.9	91.0	77.8	65.7	76.9	77.5
	HRNet-W32	384 × 288	56.1 M	21.0	72.8	**91.5**	80.7	68.7	79.3	79.7
	HRNet-W32	512 × 384	56.1 M	36.9	**74.1**	91.3	**80.7**	**69.0**	**80.1**	**80.3**
Performance Gain(R-B***)					**+2.0**	**+1.1**	**+1.1**	**+0.9**	**+1.1**	**+0.9**

**Table 5:** AP and inference speed results on COCO val. HRNet and Transpose are trained with 210 and 240 epochs, respectively, whereas Cofopose is trained with 50 epochs. Our network achieves a competitive speed/accuracy trade-off.

Method	AP	Inference Speed (FPS)
HRNet-W48	73.3	27
HRNet-W32	72.5	28
TransPose-H	74.2	38
Cofopose	74.2	36

## Data Availability

The dataset used in this paper is a public dataset.

## Abbreviations

The following abbreviations are used in this manuscript:

COCO Common Object in Context
MPII Max Planck Institut Informatik
DETR Detection Transformer
DCNN Deep Convolutional Neural Network
VATT Video–Audio–Text Transformer
HPE Human Pose Estimation
SOTA State-of-the-art
